# Transendothelial Electrical Resistance Measurement across the Blood–Brain Barrier: A Critical Review of Methods

**DOI:** 10.3390/mi12060685

**Published:** 2021-06-11

**Authors:** Judit P. Vigh, András Kincses, Burak Ozgür, Fruzsina R. Walter, Ana Raquel Santa-Maria, Sándor Valkai, Mónika Vastag, Winfried Neuhaus, Birger Brodin, András Dér, Mária A. Deli

**Affiliations:** 1 Institute of Biophysics, Biological Research Centre, 6726 Szeged, Hungary; vigh.judit@brc.hu (J.P.V.); kincses.andras@brc.hu (A.K.); walter.fruzsina@brc.hu (F.R.W.); anaraquel.santamaria@wyss.harvard.edu (A.R.S.-M.); valkai.sandor@brc.hu (S.V.); 2 Doctoral School of Biology, University of Szeged, 6720 Szeged, Hungary; 3 Department of Pharmacy, University of Copenhagen, 2100 Copenhagen, Denmark; burak.ozgur@sund.ku.dk (B.O.); birger.brodin@sund.ku.dk (B.B.); 4 In Vitro Metabolism Research, Division of Pharmacology and Drug Safety, Gedeon Richter Plc., 1103 Budapest, Hungary; m.vastag@richter.hu; 5 Center for Health and Bioresources, Competence Unit Molecular Diagnostics, AIT—Austrian Institute of Technology GmbH, 1210 Vienna, Austria; Winfried.Neuhaus@ait.ac.at

**Keywords:** blood–brain barrier, cell culture insert, electrodes, endothelial cell, epithelial cell, impedance, lab-on-a-chip, transendothelial electrical resistance, viscosity

## Abstract

The blood–brain barrier (BBB) represents the tightest endothelial barrier within the cardiovascular system characterized by very low ionic permeability. Our aim was to describe the setups, electrodes, and instruments to measure electrical resistance across brain microvessels and culture models of the BBB, as well as critically assess the influence of often neglected physical and technical parameters such as temperature, viscosity, current density generated by different electrode types, surface size, circumference, and porosity of the culture insert membrane. We demonstrate that these physical and technical parameters greatly influence the measurement of transendothelial electrical resistance/resistivity (TEER) across BBB culture models resulting in severalfold differences in TEER values of the same biological model, especially in the low-TEER range. We show that elevated culture medium viscosity significantly increases, while higher membrane porosity decreases TEER values. TEER data measured by chopstick electrodes can be threefold higher than values measured by chamber electrodes due to different electrode size and geometry, resulting in current distribution inhomogeneity. An additional shunt resistance at the circumference of culture inserts results in lower TEER values. A detailed description of setups and technical parameters is crucial for the correct interpretation and comparison of TEER values of BBB models.

## 1. Introduction

The blood–brain barrier (BBB) represents the tightest biological barrier within the cardiovascular system, which plays a crucial role in the protection of the central nervous system [1]. The interendothelial tight junctions of brain microvessels create the basis of this physical defense system which restricts the movement of ions through the paracellular space, thus contributing greatly to the development and maintenance of the ionic homeostasis in the brain [2]. This ionic tightness of the BBB can be characterized by the measurement of electrical resistance across the wall of brain microvessels [3] or culture models of the BBB using two-compartment setups [4,5]. The first measurements on the electrical resistance of brain surface microvessels were made in the early 1980s [3] and were soon followed by measurements on brain endothelial cell cultures [6], i.e., in vitro models of the BBB. In the following 40 years, great progress and an even greater diversification of the BBB culture models could be witnessed. In addition to primary brain endothelial cell- and cell-line-based systems, stem-cell-derived BBB models were introduced and have gained popularity rapidly, as reviewed recently [7]. The major technical advances in the measurement of transendothelial electrical resistance/resistivity (TEER) of BBB models include the introduction of cell culture inserts, electrodes, and instruments from commercial sources. While the culture inserts provide static conditions, the hollow-fiber cartridges [8] and, in the last 9 years, lab-on-a-chip microfluidic devices provide a dynamic environment for BBB models [9,10], providing more physiological conditions but also presenting new technical challenges.

TEER as a physical parameter is key to characterize the barrier tightness of the BBB in vitro models [4,5]. According to a large body of barrier tightness data measured on BBB culture models, both TEER and permeability for paracellular markers, biological factors influencing the tight junctions of cultured brain endothelial cells, are the most important determinants of ionic permeability across BBB models, as detailed in several works [4,7,11,12,13,14]. These biological factors include cell sources and types (primary or stem-cell-derived cells, immortalized cell lines, brain microvessels from different species), passage numbers, culture media and supplements, factors inducing BBB properties and/or elevating BBB tightness (second messengers, cytokines, mediators, receptor agonists or antagonists), culture types (mono- or different coculture settings), and culture conditions (static vs. dynamic setups with fluid flow generated shear stress). The present review does not detail these aspects of the barrier tightness of BBB culture models, which were well covered in other papers [4,7,11,12,13,14].

The aim of the present review is to describe the setups, electrodes, and instruments that are used to measure TEER across brain microvessels and in culture models of the BBB, as well as critically assess the influence of often neglected physical and technical parameters such as temperature, viscosity, current density generated by different electrode types, surface size, circumference, and porosity of the culture insert membrane.

## 2. Electrical Resistance Measurements: Principles, Methods, and Influence of Physicochemical Parameters

### 2.1. Electric Circuit Analysis of Biological Barriers

**Figure 1:**
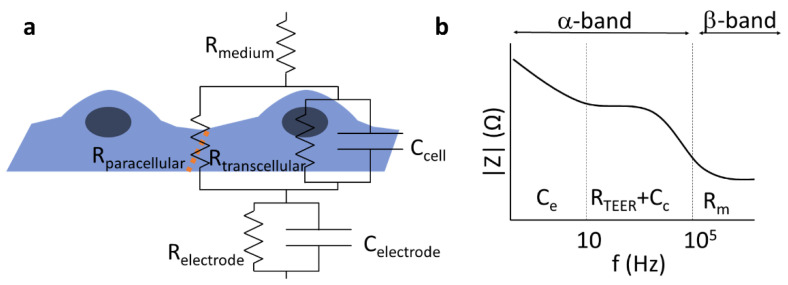
Components of the total electrical impedance measured across brain endothelial cells modeling the blood–brain barrier. (**a**) The equivalent circuit of a cell monolayer. The transendothelial resistance (R_TEER_) is primarily dominated by the paracellular resistance (R_paracellular_). In the case of electrical impedance measurement, the transcellular pathway (R_transcellular_, C_cell_) and the properties of the electrode (R_electrode_, C_eletrode_) also affect the recorded signal. The orange dotted line depicts tight junctions between brain endothelial cells. (**b)** Schematic impedance spectrum of a cell monolayer. The α-band contains the electrode polarization, the resulting resistance of the layer (R_TEER_, determined by the para- and transcellular resistances: R_paracellular_ and R_transcellular_), and the capacitance of the cell layer (C_c_), while the β-band is dominated by the resistance of the medium (R_m_) and the resulting capacitance of the system. Modified from [5].

The passive electric properties of biological barriers are determined by their mesoscopic organization including tightness of cell–cell connections, cell morphology, and membrane robustness. Hence, electric impedance measurements have been proven useful in 2D or 3D cell culture models of specific biological barriers including insert [5] or lab-on-a-chip models [15,16]. The architecture of cells—thin lipid membranes considered as fairly good insulators, surrounded by mobile ions of the electrolyte representing a relatively high conductivity at physiological salt concentrations—predestines the use of resistor–capacitor (RC) substitution-circuit models [5] to characterize the electric properties of biological barriers (Figure 1). For details, see Figures S1–S3 (Supplementary Materials).

Depending on the frequency range analyzed, information can be gained about various features of the barriers, which may be associated to specific physiological functions. The TEER of BBB culture models is primarily determined by the tight interendothelial junctions [4]; however, in other biological barriers, active ion channels can contribute to the measured resistance [17]. Below, we give a short overview of the related principles and methods used to study the TEER of BBB in vivo and in vitro.

### 2.2. Electric Impedance Spectroscopy

Electric impedance spectroscopy (EIS) can be especially useful for leaky/low-resistance models of biological barriers where modeling of the recorded impedance spectra gives information about morphological changes of cells and the cellular organization of the tissue [5]. If an electric field is applied to the sample, the mobile ions inside and outside of the cells are displaced, typically alongside the highly charged membrane surfaces, resulting in a polarization of the cells, as well as the whole sample. Electric polarization is directly related to dielectric permittivity; hence, the method is also often cited as dielectric spectroscopy. Since charge displacements are inevitably accompanied by energy dissipation, the sample can be characterized by a complex dielectric permittivity, where the real part describes the polarization, while the imaginary part describes the dissipative properties. In electronic terms, the former corresponds to the capacitive properties, while the latter corresponds to resistive properties of the sample (Figures S1–S3, Supplementary Materials). The times necessary for the polarization of cells or components representing other hierarchical levels, such as cell associates or cell organelles and macromolecules, are characterized by time constants depending on the size of the given component. Conventionally, three ranges of the dissipation time constants are distinguished [18]: the α-band (between the mHz and kHz range) is associated with electrode polarization and counterion relaxations around tissues or cells, the β-band (between ~100 kHz and 100 MHz) is characteristic of transmembrane capacitive currents (Figure 1), and the γ-band (100 MHz–100 GHz) can be assigned to the polarization of macromolecules or the reorientation of water molecules. The contribution of the different factors to the overall impedance can be distinguished by the mathematical analysis of an extended frequency range. There is an interesting technique called two-path impedance spectroscopy [19], which allows the decomposition of R_TEER_ into R_transcellular_ and R_paracellular_ terms, under certain conditions. The method is based on an EIS measurement combined with a Ca^++^-dependent modulation of R_paracellular_, as well as the determination of the permeability of a paracellular marker that is supposed to vary inversely to R_paracellular_, without affecting R_transellular_.

There are several commercially available systems for EIS, including the ECIS (Electric Cell-Substrate Impedance Sensing; Applied BioPhysics) and xCELLigence (Agilent, USA) devices. The cells are grown on a solid support containing the integrated planar gold electrodes in both setups. The main difference between the two systems is that ECIS displays impedance spectra, while xCELLigence reports a dimensionless parameter called cell index, CI = Z_n_ − Z_0_, where Z_n_ is the impedance at time point *n* and Z_0_ is the impedance without cells. These methods allow the detection of small changes in morphology, strength of adhesion, and cell–cell interactions [20]. Another EIS based setup is the cellZscope system (NanoAnalytics, Germany), designed for measuring parameters of cell monolayers grown on culture inserts (Figure S4, Supplementary Materials). In this setup, the cell monolayer covering the porous membrane of the insert is between a pair of electrodes [5]. The device automatically measures the impedance spectra and performs the mathematical analysis of the recorded signal. In addition to resistance, capacitance data are acquired. Increased cell layer capacitance due to changes in cell membrane properties can indicate cellular toxicity or dysfunction in culture models of the central nervous system barriers [21,22]. To characterize the tightness of culture models of biological barriers, however, low-frequency methods are the most widespread techniques [5].

### 2.3. Direct Current or Quasi-Direct Current Methods

The DC or quasi-DC methods are based on the measurement of ohmic resistance of the biological barriers, and they have been applied for measurements on microvessels in living tissues and brain endothelial monolayers in culture conditions. This transendo/epithelial electric resistance, measured in ohmic units, is also called “TEER”; however, in this paper, we denote it by R_TEER_, so as to distinguish it from the endothelial/epithelial surface area-corrected TEER values that represent a resistivity, measured in Ω·cm^2^ units. For details, see Section 4.2.

**Figure 2:**
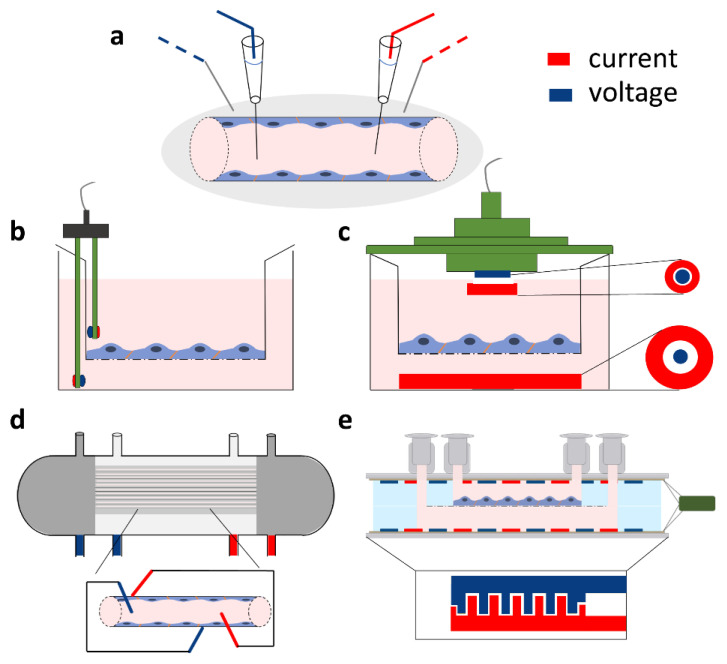
Direct current or quasi-direct current methods for in vivo and in vitro transcellular electrical resistance and impedance measurements. All types of setups include a four-point measurement method based on two current electrodes (red) and two voltage electrodes (blue). (**a**) Setup for electrical resistance registration of brain surface microvessels. (**b**) Chopstick and (**c**) chamber silver/silver chloride (Ag/AgCl) electrode arrangement for the measurement of transcellular resistance across cellular monolayers cultured on different sizes of cell culture inserts. (**d**) Impedance measurement setup on a hollow-fiber cartridge model. (**e**) Resistance measurement on a novel lab-on-a-chip model based on electrodes prepared with gold sputter coating (method described in [14]).

In animal studies the electrical resistance of brain surface microvessels is determined similarly to the electrical resistance of the axonal membrane [23]. The four-point measurement system with capillary electrodes is inserted to the brain tissue and the microvessel (Figure 2a). The intracapillary potential (V(x)) is related to the potential at the tip of the current electrode (V(0)) via the following equation:

V(x) = V(0)e^−x/λ^ (1)

where x is the distance to the measuring voltage electrode, and λ is the length constant [3,23]. The resistance of the vessel can then be calculated from the length constant of the potential decay, the vessel radius, and the blood resistivity [3]. In culture studies, based on brain endothelial cell monolayers in mono- or co-culture conditions [4,7,12], the most straightforward approach is to place one electrode in the top fluid compartment and another one in the bottom compartment (Figure 2a,b). The two compartments are separated by the cell monolayer(s) grown on a porous membrane. The resistance is calculated on the basis of Ohm’s law by taking the ratio of voltage and current [16]. However, the impedance of the electrode–electrolyte interface usually seriously compromises the measurements at DC or low frequencies, when two electrodes are used. In order to overcome the problem of electrode polarization, the four-electrode method is applied for biological samples, where second-order conductors (electrolytes) are part of the system [24]. In this case, a pair of electrodes is used to inject constant current through the sample, and another pair of electrodes measures the generated voltage across the sample (Figure 2; Figures S1–S3, Supplementary Materials). Since, in this case, there is no current flowing through the voltage electrodes, they can accurately measure the voltage drop on the sample (Figures S1–S3, Supplementary Materials). The resulting resistance of the sample (R_TEER_ + R_medium_ + R_electrode_) is calculated from the constant current of the current generator and the voltage measured by the voltage electrode pair, according to Ohm’s law. (Note that R_electrode_ can be considered negligible well above the decomposition potential of the solution.) In practice, low-frequency square waves are used instead of DC, in order to avoid gas evolution on the surface of the electrodes. The commercially available and widely used EVOM instrument (World Precision Instruments, USA) applies a 12.5 Hz square waveform. The range of the device is from 0–9999 Ω with a resolution of 1 Ω. This four-point measurement configuration is compatible with different kinds of electrode geometries as shown on Figure 2b,c,e.

The most commonly used electrode type in models of the BBB using a culture insert setup is the so-called chopstick electrode which can be easily handled to quickly measure TEER in parallel samples (Figure 2b). The current passing electrodes are made from silver and the voltage measuring electrodes are silver/silver chloride (Ag/AgCl) electrodes. During measurement, one of the sticks is placed in the top and the other in the bottom compartment containing culture medium. The placement of the chopstick electrode needs extra attention as it can result in resistance measurement inaccuracies as compared to measurements with other devices, as discussed later. As an alternative to chopstick electrodes, chamber electrodes are available (Figure 2c). The culture insert can be placed in the chamber and covered with a cap. Both the chamber and the cap contain a pair of integrated electrodes: a centrally positioned Ag/AgCl for voltage measurement and an outer, concentrically placed silver current passing electrode. Compared to the chopstick electrode, the chamber has not only fixed positions which decrease the error of random electrode positioning, but also planar electrodes with large surfaces.

Another system that is used for in vitro modeling of the BBB is the hollow-fiber model (Figure 2d). The hollow-fiber cartridges contain a bundle of capillaries made from porous membranes which mimic the geometry of blood microvessels. By growing brain endothelial cells in the lumen of these capillaries and introducing flow of the culture medium, the system can be used as a dynamic model of the BBB [25]. The capillaries are embedded in a plastic housing, resulting in an outer compartment representing the extracapillary space. There are commercially available hollow-fiber models (Flocel, Cleveland, OH, USA) with integrated TEER measurement [8]. For these, four electrodes are usually installed, two at the beginning and end of the cartridges in the extracapillary space and two within the lumen of the capillaries (Figure 2d). The impedance is then measured and averaged; then, at a defined frequency, the TEER is determined.

In the last 9 years, several types of lab-on-a-chip (LOC) devices have been developed to model the BBB (for reviews, see [9,10]). Many of these devices allow the measurement of TEER with the integration of electrodes. The general structure of LOC devices is built up by two overlapping channels separated by a porous culture membrane, i.e., the layer structure and the topology of most chip devices do not differ from those of transwell inserts; hence, their equivalent circuit is the same. Of course, the values of the R and C components do depend on the actual geometry. The electrodes for the electrical measurements are placed in the top and bottom compartments (Figure 2e). The main differences between LOCs include the channel size, the size of the overlapping region/culture surface, the electrode geometry, and material [9]. The electrodes can be wires, plated or sputter coated metal layers, or indium tin oxide (ITO) glass. The wires are inserted in the channels; the material can be Ag/AgCl as the measuring electrodes and silver as the current electrode or platinum [15]. The advantage of the wire electrodes is that they do not need special laboratory equipment for their production. The drawback of the wire-type electrodes is that they, similarly to the chopstick case, create a nonuniform current profile; thus, they suit only smaller culture surfaces. In contrast, plated or sputter-coated metal electrodes in a four-electrode setting [14,26,27], especially in an interdigitating format (Figure 2e), provide a more homogenous sensitivity distribution [28] and, therefore, a more reliable TEER measurement.

### 2.4. Effects of Physical and Physicochemical Parameters on Electrical Resistance

While biological factors, such as brain endothelial cell type, passage number, coculture, culture medium composition, and barrier-inducing factors [29], greatly influence TEER values of BBB models, as reviewed earlier [4,12], the present overview focuses on the effects of physical and physicochemical parameters on TEER measurements.

When the electrical properties of brain endothelial cell monolayers or microvessels are measured, the properties of the periphery are also included in the results (Figure 1). The resistance of the medium (in vivo: blood, extracellular fluid; in vitro: culture medium), as well as the culture membrane of inserts, and the resistance and capacitance of the electrodes have to be excluded. Thus, these parameters have to be measured before the experiment to determine the blank resistance. In the case of culture models, inserts without cells should be used to measure this background resistance. It is very important to coat the membrane in a similar way as for cells, and at least a 30-min equilibration time is needed in complete culture medium before measurements. The blank value has to be subtracted from the measured resistance to receive the resistance of the microvessel or cell layer.

Temperature has a key importance in TEER measurement, since conductivity exhibits a linear relationship with the ion mobility (Nernst–Einstein equation). On the other hand, the ion mobility in an electrolyte exhibits an exponential type of relationship with the temperature [30]. This means that the TEER of BBB culture models is measured either inside the culture incubator or at room temperature. The advantage of chamber electrodes, cellZscope, and chip devices with continuous TEER monitoring is that they allow measurements in incubators. For hand-held chopstick electrodes, the measurement is made outside the culture incubators and often at room temperature. To avoid any temperature-based fluctuations, heating pads set to 37 °C are used for quick measurements, or a temperature equilibration period is used before monitoring TEER. The equilibration time depends on the volume of the fluid compartments and the material of the culture insert. In general, this time interval is between 5 and 40 min which should be determined for individual laboratory conditions. However, a longer equilibration period may compromise the barrier function of the endothelial cell layers due to temperature drop and also lead to a shift in the pH of the bicarbonate-based culture medium to more alkaline. Another possibility is the mathematical correction of the measured TEER values [31]. The correlation between temperature and TEER is measured, and an empirical formula is determined. This method saves the time of equilibration and eliminates temperature-caused fluctuation between separate experiments; however, the calibration has to be performed for each device and insert type, as well as formatted to make it compatible for a wider use. To avoid the problems related to temperature variations, a heating pad set to 37 °C can be placed under the culture plates or chamber electrodes.

The viscosity of the culture medium also influences the TEER of BBB models by decreasing the ion mobility in the system [30], thus increasing the TEER. In static BBB models, the widely used culture media show a viscosity close to that of isotonic saline (~1 mPa·s), and only its temperature dependence is expected to influence TEER to a moderate extent, as described above. In dynamic BBB models that mimic the dynamic viscosity of blood (3–4 mPa·s) by adding colloids (e.g., 3.5% dextran) to the culture medium [32], other effects may occur, mainly due to the increased shear stress and osmotic pressure exerted by the viscosity-increasing additives. Hydroxyethyl starches (HES) are used as blood volume expanders in patients with severe blood loss. The dynamic viscosity of a 6% HES solution (HES 130/0.4, Volulyte^®^, Fresenius Kabi, Germany) is 2.5 mPa·s [33]. In a recent study, the effect of HES 130/0.4 was tested on a BBB culture model, and a barrier-tightening effect was found according to marker molecule permeability and tight junction protein expression [34].

**Figure 3:**
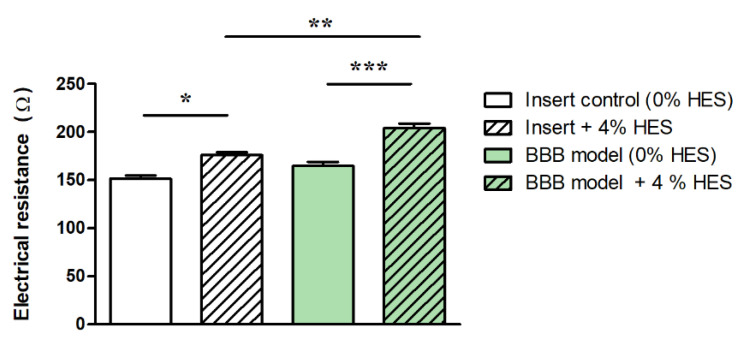
Electrical resistance of immortalized mouse cerebellar capillary endothelial cells (cerebEND) after hydroxyethylstarch (HES) treatment. Transparent columns: cell culture inserts without cells. Green columns: cell culture inserts with the cerebEND cell line modeling the blood–brain barrier (BBB) in vitro. The control groups contained 0% HES. Data are means ± SEM, *n* = 6–13, two-way ANOVA with Bonferroni post-test; * *p* < 0.05, *** *p* < 0.01, *** *p* < 0.001. Modified from [34].

In the presence of 4% HES, the resistance of cell culture inserts (without cells) increased by 16%, while it was elevated by 24% in the case of the BBB model (Figure 3). Our data indicate that higher culture medium viscosity can directly increase TEER values of BBB models.

## 3. In Vivo Measurements

**Figure 4:**
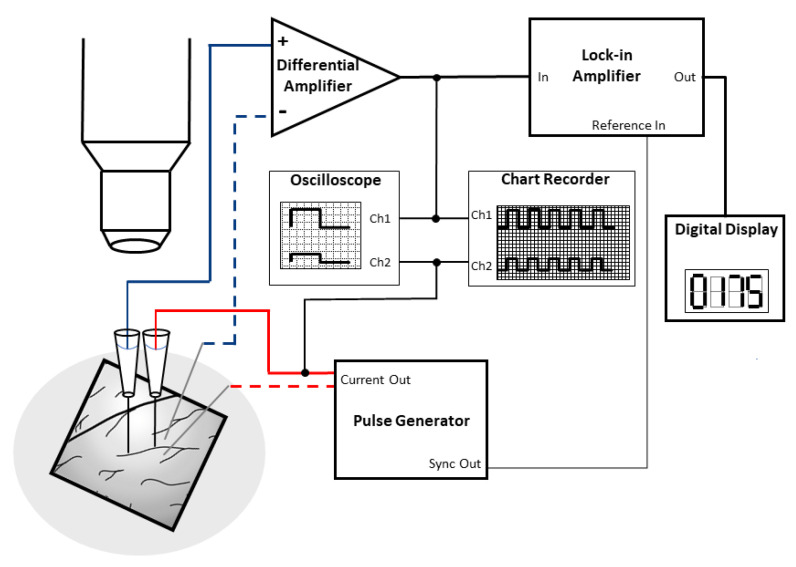
Cartoon explaining the in vivo electrical resistance measurement method of brain surface microvessels. On the left side, a cranial window allows the exposure of surface vessels for the measurement. From the two electrode pairs, one is for current injection (red), while the other one is for the potential measurement (blue) with rectangular pulses. The glass microelectrodes are filled with KCl buffer. Recordings are helped by a lock-in amplifier and an oscilloscope. Electrode insertion is controlled by precision observation using a microscope. Modified from [3].

The very low ionic permeability of the vertebrate BBB has been known for many decades, and its main role is to regulate and maintain the ionic homeostasis of the central nervous system for proper neuronal function [1]. There are few in vivo studies describing the electrical resistance of microvessels in the central nervous system, which were measured on the microvessels of the pia mater, covering the brain surface of frogs and rats. The first direct measurement of the electrical resistance of pial microvenules was performed on anesthetized frogs at room temperature [3]. In the experimental setup (Figure 4), glass microelectrodes were used (Figure 2a), which utilized two electrical circuits, one for current injection (0.1–1 µA) and one for potential measurements, with a rectangular pulse of 2–2.5 Hz (200 ms).

The average TEER of frog venous capillaries and small venules at the brain surface (Table 1) was 1870 Ω·cm^2^ in the first experimental setup [3] and ~2000 Ω·cm^2^ in later studies [35,36].

A similar technique was used to measure the electrical resistivity of brain surface arterial and venous microvessels in anesthetized rats at 37 °C (Table 1). The average electrical resistivity of arterial microvessels was 1490 Ω·cm^2^, while, in venous vessels, a lower value of 918 Ω·cm^2^ was measured [37]. Individual TEER data varied between 500 and 5900 Ω·cm^2^ during the measurements. In a subsequent measurement series, TEER values in the same range were obtained [38]. In vivo microvessel resistance measurements are highly complex and technically challenging, partly due to the need for precision surgical expertise. This might be the reason why the method has not been widely adapted and no new data have been reported in the last 30 years. Another critical point is that the pial microvessels at the brain surface may be different from parenchymal ones [39]. Due to the lack of direct measurements, only assumptions can be made regarding the electrical resistance of human capillaries in the brain parenchyma.

## 4. Measurements on Insert Models

Adapting a technique used in epithelial cell biology, the first cell culture inserts for BBB studies were custom-made from nylon mesh and polycarbonate tubing [40]. At present, a wide variety of inserts are available using different membrane materials with different pore size, pore density, and membrane surface from commercial sources. This diversity of the inserts, as well as measurement devices and electrodes, results in the heterogeneity of TEER results even when the same barrier models are used. Here, we discuss the technical and physical aspects focusing on measurement devices, electrodes, insert membrane surface size, circumference, and porosity, as factors influencing TEER data.

### 4.1. Influence of Measuring Devices: Differences between Chopstick and Chamber Electrodes and the Cellzscope System

**Figure 5:**
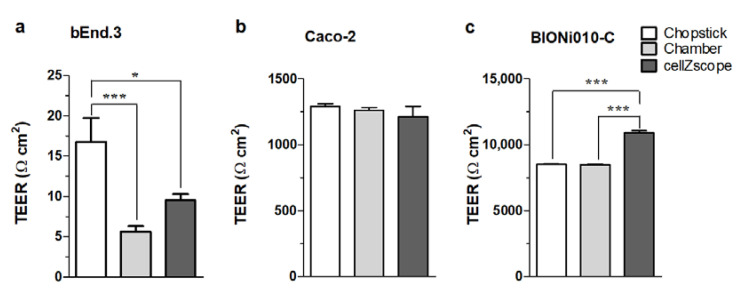
Transcellular (endothelial or epithelial) electrical resistivity (TEER) measurements on barrier models expressing different tightness: (**a**) bEnd.3 cells represent a model with low barrier tightness, (**b**) the Caco-2 intestinal cell line serves as a tight epithelial monolayer, and (**c**) the Bioni010-C derived human endothelial cells express exceptionally tight barrier properties. Data are shown as means ± SEM of one individual passage with 12 individual permeable supports (*n* = 1, total *N* = 12) for Caco-2 and Bioni010-C derived endothelial cells, and one individual passage with 24 individual permeable supports (*n* = 1, total *N* = 24) for bEnd-3 cells. Statistical analysis one-way ANOVA analysis and Tukey’s multiple comparison test; * *p* < 0.05, *** *p* < 0.001.

The use of different TEER measurement devices can lead to different TEER values even when using the same culture models, which can make it difficult to compare TEER results among different laboratories. The different measurement devices each have their strengths and weaknesses. In the chopstick electrode setup, one electrode pair is positioned in the upper chamber, while a second electrode pair is positioned in the lower chamber between the polarized cell monolayer (Figure 2b). The chopsticks can easily be moved from one culture insert to another without disturbing the cell layers too much. Contrarily, each insert must be transferred from the culture plate into the chamber electrode for recording TEER in the EndOhm (World Precision Instruments, Sarasota, Florida, USA) or the cellZscope system. The cell monolayer can be disturbed by the transfer, as well as temperature and pH changes, which can potentially result in large fluctuations during TEER measurements. However, the reproducibility of measurements with chopstick electrodes can also be a serious concern due to large variations in electrode positioning, the small electrode surfaces, and their geometry related to the membrane. To compare how measuring devices influence TEER values, three TEER measurement devices (chopstick and chamber electrodes, and a cellZscope system) were used to assess TEER values across three different cell culture models: bEnd.3 mouse brain endothelial cell line [41], Caco-2 human epithelial cell line [42], and human endothelial cells derived from Bioni010-C induced pluripotent stem cells [43]. The three types of cell culture models represent (1) low barrier tightness (TEER <25 Ω·cm^2^), (2) medium-to-high barrier integrity (TEER ~1000–1500 Ω·cm^2^), and (3) extremely tight barrier properties (TEER >5000 Ω·cm^2^), respectively (Figure 5). TEER measurement was randomized, and each insert was measured with all three devices in a random order as described in the Supplementary Materials (Table S1). All cell types, devices/electrodes, and TEER values are shown in Table S2 (Supplementary Materials).

No differences were observed in the TEER measured in Caco-2 cells using the three different methods regardless of the order of TEER measurement (Figure 5b). For Bioni010C-derived endothelial cells that represented a cell model with an extremely tight barrier, cellZscope measured a significantly higher TEER as compared to the TEER monitored by chamber or chopstick electrodes (Figure 5c). The chamber and chopstick electrodes measured a similar TEER (Figure 5c). The TEER measurements in bEnd.3 cells showed that this cell model formed a leaky barrier with low tightness. Notably, the average TEER values were different using the three different devices in this low-TEER range (Figure 5a). We observed a significantly higher average TEER using the chopstick electrodes as compared to the two other devices. This is likely due to the inhomogeneous electrical field when using chopstick electrodes that leads to a systematic overestimation of TEER.

**Figure 6:**
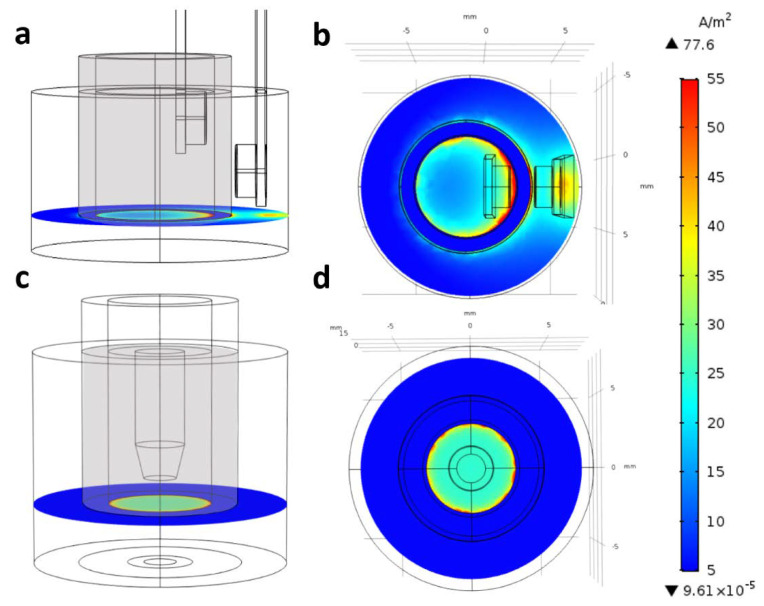
COMSOL modeling of electrical resistance measurement across culture inserts (diameter: 6.5 mm, 24-well type) with (**a**,**b**) chopstick (STX2) or (**c**,**d**) chamber electrodes. The thin black lines show the borders of the objects and their components (borders of the electrodes on the surface), while the grid behind is to indicate the actual size of the objects. For easier understanding, the optically transparent cylindrical wall of the inserts is depicted in gray. The current density was calculated and is shown at the level of the cell layer at the bottom of culture inserts.

To prove this hypothesis, we modeled the electric current density created by chopstick and chamber electrodes in culture inserts (Figure 6; Figures S5 and S6, Supplementary Materials). In Figure S6 (Supplementary Materials), the plots show the current density in streamline representation; denser lines denote higher current density in that region. We can see that, for the chopstick electrodes, the electric current should go around the blocking (insulator) cylindrical wall of the inserts; hence, the conductivity of the cell layer close to the electrodes contributes more than the other, farther parts (Figure S6a, Supplementary Materials). In the chamber electrode configuration, the current goes directly through the cell layer, and, due to the axial symmetry of the planar electrodes with larger surface than in the case of the chopstick ones, the current density is relatively even for the whole cell layer (Figure S6b, Supplementary Materials). These differences in the current density are clearly visible, as shown in the plane of the cell layer at the bottom of the cell culture inserts (Figure 6). Chopstick electrodes create a strongly inhomogeneous electric field (Figure 6a,b), while chamber electrodes create a more uniform current density (Figure 6c,d). The presented calculation refers to culture inserts with 6.5 mm diameter (inserts fitting 24-well plates). Our data are in agreement with the calculation of Yeste et al., who modeled sensitivity distribution when TEER was measured in culture inserts with 6.5 and 12 mm diameter using chopstick electrodes [28]. They also found for the chopstick electrode that zones close to the electrodes contributed more than zones at a greater distance. This implies that the sensitivity field in culture inserts changes with their surface area; in 12 mm culture inserts at low TEER values (10 Ω·cm^2^), the sensitivity field over half of the membrane area was less than 25% of the optimal sensitivity.

Another important finding was that the sensitivity field depended on the TEER range; a higher TEER led to a more uniform sensitivity distribution. The sensitivity inhomogeneity was large at TEER ≤100 Ω·cm^2^, while sensitivity differences were less than 5% for inserts with 6.5 mm diameter and 23% for 12 mm inserts when TEER was ≥1000 Ω·cm^2^ [28]. These observations may explain the TEER differences measured on 12 mm inserts with chopstick and chamber electrodes at a low-TEER range (<20 Ω·cm^2^) as compared to high-TEER ranges (Figure 5). Regarding the TEER differences found between measurements across the tightest cellular barrier (Figure 5c), these might not be related to the electrodes, but rather to the measuring instruments. The EVOM uses a fixed frequency, while the cellZscope measures impedance spectra in a wide range of frequency and calculates the TEER depending on the tightness of the model. Therefore, the position of the TEER “plateau” within the α-band can be dissimilar for models representing very different tightness (Figure 1b), which may be the reason for the higher TEER values in the tightest biological model (Figure 5c).

### 4.2. Effect of the Insert Circumference

Raw data of transmembrane resistance measurements are obtained in ohmic units, which, however, inversely depend on the area of cross section of the medium at the barrier layer.

R_TEER_ = ρ·l/A, (2)

where RTEER is the transmembrane resistance, ρ is the volumetric specific resistivity, l is the thickness, and A is the area of the barrier layer. In order to account for this dependence, the measured resistance values are multiplied by the surface area of the filter membrane, resulting in a sort of specific resistivity (ρ·l) of the barrier layer, assuming that the resistivity of the filter membrane and the solution are negligible or corrected for. These modified values, measured in Ω·cm^2^ units, are supposed to be comparable between experiments carried out with arbitrary size of cell growth area; thus, they are considered as the conventional representation of TEER: TEER = R_TEER_ A.

However, Yeste et al. highlighted a possible source of overcompensation in such TEER values, stemming from the inhomogeneity of the electric field applied to measure the resistance of the sample [28]. The deviation from the ideal case is especially emphasized when chopstick-type electrodes (see also Figure 2b), low resistivity, and large membrane areas are used. Based on COMSOL simulations, they introduced a geometric correction factor (GCF) to be applied as a multiplicator on the conventionally derived TEER values. Although they did not provide explicit data for chamber electrodes, the results of our simulations imply that the error due to the inhomogeneity of the electric current density used in TEER measurements should be considerably less for chamber than for chopstick electrodes (Figure 6; Figure S6, Supplementary Materials).

**Figure 7:**
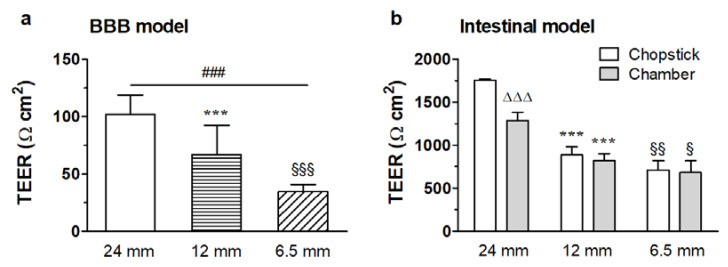
Effect of the cell culture insert circumference on the transendothelial electrical resistivity (TEER). (**a**) TEER measured with chopstick electrodes on immortalized mouse cerebellar capillary endothelial cells (cerebEND) using different sizes of cell culture inserts. Means ± SEM, *n* = 15–76, one-way ANOVA with Bonferroni post-test; ^###^ *p* < 0.001, between 24 mm and 6.5 mm cell culture inserts; *** *p* < 0.001, between 24 mm and 12 mm cell culture inserts; ^§§§^ *p* < 0.001, between 12 mm and 6.5 mm cell culture inserts. (**b**) TEER measured on an intestinal barrier model on different sizes of cell culture inserts both with the chopstick and the chamber electrode setup. Means ± SEM, *n* = 6, two-way ANOVA with Bonferroni post-test; ^ΔΔΔ^ *p* < 0.001, between chopstick and chamber electrodes; *** *p* < 0.001, between 24 mm and 12 mm cell culture inserts; ^§^ *p* < 0.05, ^§§^ *p* < 0.01, between 12 mm and 6.5 mm cell culture inserts.

Selecting some typical series from our database, where TEER values were determined by both chopstick and chamber electrodes, it can be seen that, even for relatively tight barriers (near 1000 Ω·cm^2^), chopstick data systematically overestimate TEER values as compared to values measured by chamber electrodes (Figure 7b). However, after correcting the former with the relevant GCF factors from Yeste et al., the corresponding values of the two series coincide within 3% for 6.5 mm and 12 mm filter diameters, suggesting that data measured by chamber electrodes in this high-TEER range are apparently free from the error stemming from field inhomogeneity. Although Yeste et al. did not provide GCF data for the six-well filter size (D = 24 mm), it is reasonable to assume that TEER_chamber_/TEER_chopstick_ gives a good estimate for the GCF correction for the entire filter size range used in scientific practice, while also giving experimental support to the theoretical results by Yeste et al. [28].

On the other hand, the remaining dependence of the chamber electrode data and the GCF-corrected chopstick values of TEER on the culture insert size implies that there are still additional factors distorting TEER measurements. In order to interpret the systematic dependence of the GCF-corrected TEER (TEER_c_) on the insert size, we assume that the barrier layer near the wall of the insert is ”leaky”. In other words, not only the measuring electric field, but also the barrier tightness is inhomogeneous. The leak can be modeled as a sort of parasitic shunt resistance, coupled parallelly with the actual resistance of the barrier layer [44]. Due to geometric reasons, the shunt resistance (R_d_) is inversely proportional to the circumference of the filter (D·π, where D is the insert membrane diameter) and a narrow width (d).

R_d_ = ρ_d_·l/D·d·π, (3)

where d and ρ_d_ are assumed to be invariant to D, and d << D. Elementary calculation yields the following expression:

TEER_c_ ≈ TEER_r_/(1 + (d/D)·(ρ/ρ_d_)) = TEER_r_/(1 + x/D), (4)

where x = d·ρ/ρ_d_ is a diameter-independent factor. From the TEER_c_ data measured at three different D values, x and the ”real” TEER_r_ can be obtained by linear regression fitting. We determined this correction factor for relatively low- and high-resistivity barrier layers grown on 24-well format culture inserts (Figure 7a,b). From the low-resistance data, we obtained x = 0.8 and TEER_r_ = 78 Ω cm^2^, while, for the high-resistance case, we obtained x = 1.2 and TEER_r_ = 1780 Ω cm^2^. Since the correction factor is >1, such an effect results in the underestimation of the real TEER_r_ value without correction.

### 4.3. The Influence of the Membrane Porosity of Culture Inserts

The porosity of the membranes, i.e., how many pores are present per area on the membrane, can also affect the TEER data. When comparing inserts from three different manufacturers with different porosities of the same material (polyethylene terephtalate) in 24-well format, the raw electrical resistance data already showed significant differences (see Table 2).

**Figure 8:**
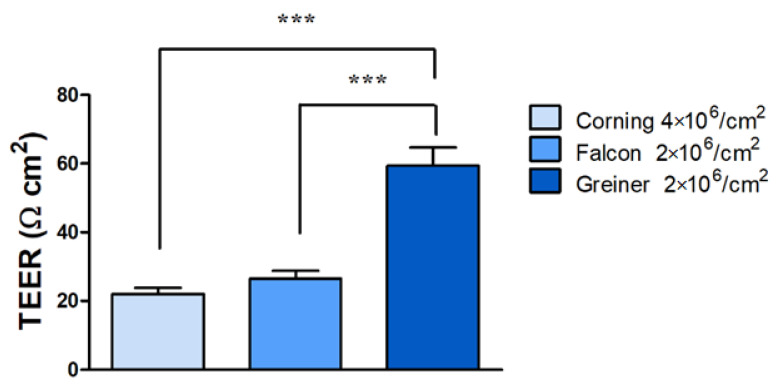
Influence of membrane porosity of culture inserts on the measured transepithelial electrical resistivity (TEER). The TR146 human epithelial cell line was cultured for 2 weeks on polyethylene therephthalate (PET) cell culture inserts from different manufacturers with 2 × 10^6^ or 4 × 10^6^ pores/cm^2^ in 24-well format. Means ± SEM, *n* = 3, one-way ANOVA and Bonferroni test; *** *p* < 0.001.

The inserts without cells in measurements over 4 weeks with the highest porosity of 4 × 10^6^ pores/cm^2^ gave the lowest TEER values (Table 2), whereas a twofold higher electrical resistivity was measured with chopstick electrodes when the pore density values were half of that (2 × 10^6^ pores/cm^2^). We tested all three types of inserts to find the optimal barrier conditions for TR146 cells (see detailed culture conditions of submerged culture in our recent paper [45]). The corresponding average TEER data with TR146 cells after 2 weeks in culture varied between 22 and 59 Ω·cm^2^ depending on the insert type (Figure 8).

Taken together, these data are a good example that the porosity of the inserts significantly affects the electrical resistance, whereby a higher pore density of the membrane leads to a lower TEER of the inserts. In addition, it should be considered that not only the porosity, but also the membrane material, even if it is the same chemical type, but from a different manufacturer, can influence the growth and barrier formation of the cell layers, which can ultimately lead to the observed different TEER values. Consequently, it is recommended to include the porosity of the membranes as a factor when TEER data are compared and interpreted.

## 5. TEER Measurements on Hollow-Fiber Cartridge Models

Although hollow-fiber models of the BBB are less widely used than the static insert models, they are valuable systems for dynamic modeling of the BBB (for a review, see [46]). The capillaries in the cartridges are connected to a tubing system that usually leads in a closed circuit to a growth medium reservoir bottle. The pumping system allows both the flow rate and the pumping rate to be adjusted in order to adapt the pulsatile flow to the aimed physiological conditions [8,25]. Accesses via luer-lock three-way valves are located both upstream and downstream of the capillaries and in the extracapillary space in the anterior and posterior areas along the pumping direction. The cells, media, and samples are introduced and drawn into and from the system via these access points. Communication between the lumen of the capillaries and the ECS is possible through the pores in the capillaries. Continuous monitoring of TEER by the four-electrode setup shown in Figure 2d can be followed online over time in the incubator. For data interpretation, several points should be considered when comparing TEER data obtained on the hollow-fiber system with TEER data measured in other models. In this system, background TEER values (blank values) measured on hollow-fiber modules without cells need to be subtracted from raw TEER data. The growth surface area in these hollow-fiber flow models is much larger than in the culture insert models. This larger surface (13.5 cm^2^ for cartridges with 19 capillaries) results in higher TEER values in the hollow-fiber flow models when compared with TEER values from other BBB models with smaller culture surfaces (0.3–4.6 cm^2^) using other measurement setups. For this reason, it is recommended to perform transport experiments with paracellular markers and molecular analyses in addition to TEER measurements when comparing different model setups. Nevertheless, it has to be underlined that a very interesting aspect of the hollow-fiber models seems to be that they can be operated for weeks up to several months, and they are, therefore, especially suitable for mid-term or long-term experiments to study chronic effects.

## 6. TEER Measurements on Microfluidic Lab-on-a-Chip Models

The commercially available TEER measuring systems require static conditions (without fluid flow) on a macroscopic scale. In addition, the mobile electrodes (chopstick or chamber) are not suitable to measure TEER in specifically designed microchannel systems [16]. The integration of the electrodes in the chip devices (Figure 2e) reduces the fluctuation of the measured signal observed when using chopstick electrodes of random electrode placement, as well as lowers the signal-to-noise ratio. If permeability assays are also to be performed in the LOC device, a relatively large (≥0.3 cm^2^) membrane surface is needed; thus, the need for planar electrodes is inevitable.

A low channel height (~100 µm) is important to set appropriate shear stress and to reduce the volume of cell culture media and reagents. In the case of low-TEER range (below 100 Ω·cm^2^), the density of the generated current can be much higher below the electrodes [28]. This results in either increased or decreased sensitivity at the nearby zones of the cell monolayer. The inhomogeneity of the current density can be compensated for by an optimized electrode design [27,28]. Alternatively, a geometric correlation factor can be applied for the proper interpretation of the measured results [28]. The current density is almost homogeneous in higher channels (>500 µm) and in the case of high TEER values (>1000 Ω cm^2^), which means that the geometry of the electrodes becomes less important in such systems or barrier types [28].

## 7. Conclusions

Physical and technical parameters greatly influence the measurement of TEER across BBB culture models. We demonstrated that different (commercially available) setups, electrodes, and even instruments, widely used to determine the ionic permeability of brain endothelial cell layers, may result in threefold differences in TEER values of the same biological model, especially in the low-TEER range. We showed that elevated culture medium viscosity, often used to increase shear stress in microfluidic models, significantly increases TEER values. Comparing four-electrode setups, TEER data measured by chopstick electrodes can be threefold higher than values measured by chamber electrodes. The differences are due to electrode size and geometry resulting in current distribution inhomogeneity. This difference can be compensated for with geometric correction factors calculated for inserts with different diameters; however, as proven through measurements and calculations, an additional shunt resistance at the circumference of culture inserts needs to be taken into account. This shunt effect results in lower TEER values, especially at measurements in the low- and middle-TEER range, as well as in the case of culture inserts with small diameters. While, for BBB models with low barrier integrity/low TEER range, the sizes of the culture inserts and electrode types are critical, for extremely tight barrier models, the selection of measuring device can influence TEER data. In contrast to devices working at fixed low frequency with four electrodes, instruments measuring impedance spectroscopy (two-electrode setup) can give higher TEER values. TEER values are multiplied with the effective cell growth area of the membranes of the culture inserts or other devices. The surface of the membranes, therefore, is a key parameter and may result in one order of magnitude difference between TEER values, as in the case of hollow-fiber models compared to culture inserts. Lastly, the pore density of the culture insert membrane, as well as inserts from different commercial sources with the same type of material can also affect TEER measurements, as demonstrated using the same barrier model and three different culture insert types. We recommend selecting the electrode type, measuring device, and culture insert size depending on the barrier properties of the BBB models, as well as using mathematical correction for certain types of electrodes and insert sizes. A detailed description of culture inserts, including membrane type, growth area, pore size and density, culture medium viscosity (if higher), electrode types, and measuring devices, is crucial for the correct interpretation and comparison of ionic permeability of BBB culture models.

## Figures and Tables

**Table 1:** Electrical resistivity (TEER) of brain surface microvessels. All TEER values are means ± SEM, except for the one marked by an asterisk (*), which is the mean ± SD.

Species/Area	Vessel Type	Diameter (µm)	TEER (Ω·cm^2^)	Current (nA)	Frequency (Hz)	Reference
Frog (*Rana temporaria*)/ Pia mater	small venules	N.D.	1870 ± 639 *	100–500	2.5	[3]
	small venules	18–47	~2000	1000		[35]
	postcapillary venules	26–74	2240 ± 90	100–1000	2	[36]
Rat (*Rattus norvegicus*)/ Pia mater	arterial mv.	10–60	1490 ± 170	50–100	2.5	[37]
	venous mv.		918 ± 127			
	pial mv.	25–50	1488 ± 213			[38]
	arterial mv.		2050 ± 316			
	venous mv.		798 ± 159			

**Table 2:** Electrical resistance dependency on insert porosity. Blank values and TEER are presented as the mean ± SD, *n* = 3. PET, polyethylene terephthalate.

Product Name	Product Number	Porosity	Surface Area (cm^2^)	Material	Pore Size (µm)	Blank Values (Ω)	TEER (Ω·cm^2^)
Corning	CLS3470	4 × 10^6^/cm^2^	0.330	PET	0.4	117.0 ± 4.4	22.0 ± 1.8
Falcon	BDL 353095	2 × 10^6^/cm^2^	0.300			211.9 ± 6.6	26.5 ± 2.3
Greiner	662641	2 × 10^6^/cm^2^	0.336			275.6 ± 3.2	59.4 ± 5.3

## Supplementary Materials

The following are available online at https://www.mdpi.com/article/10.3390/mi12060685/s1: Materials and methods for the included original data, and model calculations for current distribution, Table S1: Order of TEER measurement, Table S2: Cell culture models and measurement setups described in the paper, Figure S1: Overview of current movement in traditional two-compartment setup of a tight cell monolayer, Figure S2: A detailed schematic of the individual resistances in the two-compartment setup of a tight cell monolayer, Figure S3: Schematic representation of contributors to impedance of a cell monolayer cultured in a two-compartment setup, Figure S4: Schematic representation of the cellZscope system, Figure S5: Screenshot of the graphical user interface (GUI) of the Comsol software, Figure S6: Comsol modeling of electrical resistance measurement across culture inserts.
