# Supplementary material for: Transendothelial Electrical Resistance Measurement across the Blood–Brain Barrier: A Critical Review of Methods
Source: Micromachines (Basel). 2021 Jun 11;12(6):685. doi: 10.3390/mi12060685 (PMC8231150; doi:10.3390/mi12060685)
Supplement: Supplementary file 1 [file micromachines-12-00685-s001.zip › micromachines-1239744-supplementary.pdf]

Supplementary Materials to

Transendothelial electrical resistance measurement across the blood-brain barrier: a critical review of methods and devices

Judit P. Vigh [1,2,†], András Kincses [1,†], Burak Ozgür [3], Fruzsina R. Walter [1], Ana Raquel Santa-Maria [1,#], Sándor Valkai [1], Mónika Vastag [4], Winfried Neuhaus [5], Birger Brodin [3], András Dér [1,*], Mária A. Deli [1,*]

† these authors contributed equally to this manuscript
1 Institute of Biophysics, Biological Research Centre, Szeged, Hungary; vigh.judit@brc.hu (J.P.V.), kincses.andras@brc.hu (A.K.), walter.fruzsina@brc.hu (F.R.W.), valkai.sandor@brc.hu (S.V.), deli.maria@brc.hu (M.A.D.); der.andras@brc.hu (A.D.)
2 Doctoral School of Biology, University of Szeged, Szeged, Hungary
3 University of Copenhagen, Denmark burak.ozgur@sund.ku.dk (B.O.), birger.brodin@sund.ku.dk (B.B.)
4 In Vitro Metabolism Research, Division of Pharmacology and Drug Safety, Gedeon Richter Plc., Budapest, Hungary; m.vastag@richter.hu
5 Austrian Institute of Technology, Vienna, Austria Winfried.Neuhaus@ait.ac.at (N.W.)

# Current affiliation: Wyss Institute for Biologically Inspired Engineering at Harvard University, Boston, Massachusetts, USA; anaraquel.santamaria@wyss.harvard.edu
* Correspondence: der.andras@brc.hu (A.D.); deli.maria@brc.hu (M.A.D.)

Materials and Methods

Cell culture

cerebEND cell line

For the viscosity experiment cerebEND cells were cultured in Dulbecco´s Modified Eagle Medium (DMEM,4.5 g/l glucose) with 10% fetal calf serum (FCS) and 1% penicillin/streptomycin (P/S). The cells were incubated in a humidified incubator (95 % atmospheric air, 5% CO_2, at 37°C). Cells at a density of 4×10^4 cells/cm^2 were seeded on collagen-IV coated 12-well PET inserts from Corning (CLS3460-48EA). TEER was measured with the EVOM instrument and STX2 chopstick electrodes (World Precision Instruments Inc., USA). The treatment solutions were 0% and 4% HES solutions (Gerhartl et al., 2020) diluted in DMEM from six percent Volulyte® (0% HES = 27.5% DMEM (incl. 10% FCS+1% P/S) + 72.5% 0.9% NaCl; 4% HES = 27.5% DMEM (incl. 10% FCS+1% P/S) + 66.7% 6% HES + 5.8% 0.9% NaCl). For Figure 7a cerebEND cells at a density of 4×10^4 cells/cm^2 were seeded on collagen-IV coated PET inserts of 6- , 12- and 24-well format from Falcon® (353090, 353180, 353095).

bEnd.3 cell line

bEND.3 cells were purchased from ATCC (Manassas, Virginia, USA) and cultured in T-75 culture flasks. The cells were incubated at 37°C (95 % atmospheric air, 5 % CO_2) and passaged once a week. The cells were maintained in DMEM supplemented with 1% L-Glutamine, 100 U/ml and 100 µg/ml penicillin andstreptomycin and 10% FBS. The cells were seeded on collagen-coated (3.39 µg/cm^2) Transwell® polycarbonate membrane filter supports (12-well, surface area 1.12 cm^2, pore size 0.4 µm) with a seeding density of 2×10^5 cells per insert, and cultured for 21 days with medium three times per week.

Bioni010-C stem cell derived endothelial cells

The human induced pluripotent stem cell line, Bioni010-C, were kindly provided from Bioneer A/S (Hørsholm, Denmark) and cultivated as described in an already established differentiation protocol developed by Stebbins et al. (2016). Briefly, Bioni010-C cells were maintained on Matrigel® (354230)

coated 6-well culture plates in mTesR1. The cells were passaged every fourth day using Versene (15040–066, Thermo Fischer Scientific) for maintenance. When Bioni010-C cells reached 80% confluence, the cells were dissociated using Accutase (A1110501, Thermo Fischer Scientific). A single cell suspension in mTesR1 supplemented with 10 µM Y27632 dihydrochloride (254, Tocris Bioscience, Bristol, Great Britain) was seeded on Matrigel coated 6-well culture plates at a seeding density of 1.05 × 10^4 cells/cm^2. The cells were cultured in mTesR1 for three days, followed by medium change to UM consisting of Dulbecco's Modified Eagle's Medium/Nutrient Mixture F-12 (DMEM/F12), 1x Eagle's minimum essential medium (MEM) non-essential amino acids, 1mM L-glutamine, 0.1 mM β-mercaptoethanol and 20% knockout replacement serum (KOSR) all from Gibco ThermoFisher Scientific (Waltham, MA, USA). After five days of culture in UM, the medium was changed to EC^+ medium (containing human endothelial serum-free media (hESFM) (11111, Thermo Fisher Scientific), 1% bovine platelet poor plasma-derived serum (PDS) (50-443-029, Thermo Fisher scientific) and 20 ng/ml human basic fibroblast growth factor (bFGF) (233-FB, R&D Systems, Minneapolis, MN, USA) and 10 µM all-trans retinoic acid (RA) (R2625)) and cells were cultured for two days. Cells were then dissociated by Accutase and seeded onto 1.12 cm^2 permeable polyester membrane filter supports with a 0.4 µm pore diameter (Transwell 3460, Corning) coated with 400 µg/ml collagen IV (C5533) and 100 µg/ml fibronectin (F1141) in sterile grade water. The seeding density was 8.9 × 10^5 cells/cm^2. The medium was changed to EC^- (containing hESFM and 1% PDS) after one day of culture on permeable filter supports, and cells were ready for experiment the following day.

Caco-2 cells

The Caco-2 cells used in this study were in passage 19. The cells were maintained in T-75 culture flasks and split once a week. The cells were seeded on collagen-coated (3.39 µg/cm^2) Transwell® polycarbonate membrane filter supports (12-well, surface area 1.12 cm^2, pore size 0.4 µM) with a seeding density of 2 × 10^5 cells per insert, and cultured for 21 days (95 % atmospheric air, 5 % CO_2, 37 ^{0}C). The cell medium was Dulbecco's Modified Eagle's Medium-AQ (DMEM) supplemented with 10 % FBS, 10 µL/ml nonessential amino acids (x100), 100 U/ml to 100 µg/ml penicillin-streptomycin solution, 0.8 mg/ml geneticin, and 2 µg=ml puromycin. The culture medium was changed three times per week.

TR146 cell line

The buccal carcinoma cell line TR146 (SigmaAldrich, #10032305) was cultured in T-75 flasks in DMEM with 10% fetal calf serum and 1% penicillin/streptomycin. The cells were incubated at 37°C (95 % atmospheric air, 5 % CO_2) as described earlier (Lin et al., 2020). Seeding density of cells was 4.29 × 10^4 cells/cm^2 and 3 different inserts were used as follows: Corning (CLS3470), Falcon (BDL 353095) ThinCert Inserts (Greiner, #662610).

TEER measurements

Briefly, the cells were equilibrated with ambient room temperature for 20 minutes and TEER across cell monolayers was measured with an Endohm 12-cup electrode chamber or STX2 chopstick electrode set connected to an EVOM voltmeter (both from World Precision Instruments Inc., USA) or a CellZscope (NanoAnalytics Gmph, Münster, Germany). In case of cerebEND and TR146 cells the equilibration period lasted for 40 minutes. The electrodes were treated with ethanol for 5-10 minutes followed by a wash with deionized water (2x). The electrodes were then equilibrated in the respective cell culture medium for 15 minutes before TEER measurements. The TEER measurement devices were divided into three different "measurement groups": CellZscope = A, Cup electrode = B and Chopstick = C. This allowed us to measure the TEER of each permeable support in six different orders or measurement groups.

Table S1. Order of TEER measurement. cellZscope = A, Chamber electrode = B and Chopstick = C.

Group 1	Group 2	Group 3	Group 4	Group 5	Group 6
ABC	ACB	BAC	BCA	CAB	CBA

Each permeable filter support was randomly assigned to one of the six groups according to the randomly generated numbers between 0 and 1 using the RAND() function in Excel and TEER was measured accordingly.

Comsol simulation
For numerical calculations Comsol 5.3 was used with Electric Currents (ec) physics module. The electric current can be considered as a DC current switched on/off for several hundreds of milliseconds, therefore stationery study was chosen. For calculation, the input current was set to 1mA and the electric conductivity of cell culture medium was 1.5 S/m. The geometry of the simulation volume was defined as a cylinder of medium without the volume the electrodes occupied. The input and output of the electric current was set to the boundary surfaces corresponding to the electrodes. For the electrically insulating cylindrical wall of the cell culture insert was taken as polymethyl-methacrylate, and the corresponding physical parameters of the built-in library was used for the calculations. There were two models: the chopstick electrodes and the chamber electrodes. The results of the calculations are presented in Figure 6.

Statistical analysis
GraphPad Prism 5 software (GraphPad Software, USA) was used for statistical analysis. Data are presented as means ± SEM or SD, where means were compared using one or two-way ANOVA analysis with Bonferroni post-tests or Tukey's multiple comparison tests.

Results

Table S2. Cell culture models and measurement setups described in the paper. TEER values of the different cell lines used are also indicated in Ω cm^2.

Cell types	Electrode type or device	Insert diameter	TEER values Ω cm^2
**BBB cell lines or models**			
**cerebEND**	chopstick	6.5 mm	34.8
		12 mm	66.9
		24 mm	102.2
**bEnd.3**	chopstick	12 mm	16.8
	chamber		5.6
	cellZscope		9.5
**Bioni010-C**	chopstick		8488
	chamber		8475
	cellZscope		10885
**Epithelial models**			
**Caco-2**	chopstick	6.5 mm	710
	chamber		681
	cellZscope		1757
	chopstick	12 mm	888-1290
	chamber		821-1262
	chopstick	24 mm	1212
	chamber		1288
**TR146**	chopstick	6.5 mm	22-59

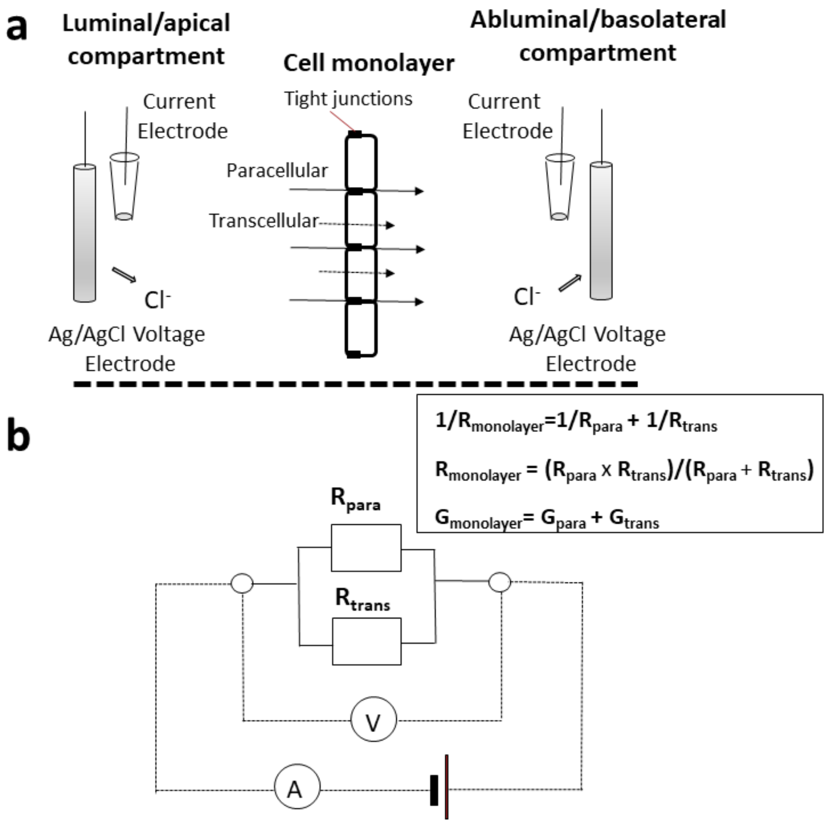

Figure S1. Overview of current movement in traditional two-compartment setup of a tight cell monolayer. **a.** Each compartment is equipped with a current electrode and a voltage electrode (often Ag/AgCl electrodes). The generated current passes the monolayer, either paracellularly via junctions or transcellularly via ion channels or intrinsic membrane conductance (very low). The voltage electrode registers the voltage at a given current, and the total resistance in the system can be calculated from Ohms law. **b.** Electrical circuit depicting the major DC circuit components. R_{para} is the resistance of the paracellular pathway, R_{trans} is the resistance of the transcellular pathway. The current source is a battery, as is the case for most traditional DC measurements (chopstick of cup-type setups), A and V are symbols for an ampere meter and a voltmeter, respectively. Assuming that the resistances in the system are linear within the voltage ranges used (an ohmic resistance), the $R_{monolayer}$ can be calculated from Ohm's and Kirchhoff's laws. Alternatively, conductances (G) can be used, simplifying the calculations. The $R_{monolayer}$ of endothelial or epithelial monolayers is called TransEndothelial (or Epithelial) Electrical Resistance, abbreviated TEER.

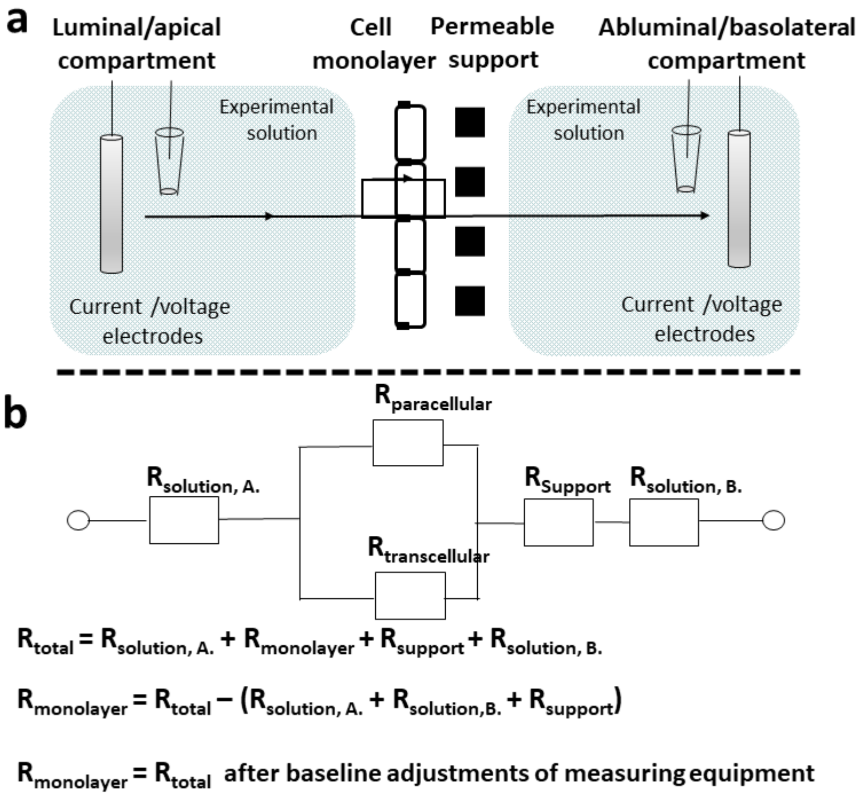

Figure S2. **A detailed schematic of the individual resistances in the two-compartment setup of a tight cell monolayer. a.** Current will pass the solutions, which will contribute to the overall resistance between the electrodes. Furthermore, the resistance of the permeable support will also contribute. **b.** A diagram showing the resistances in the system. $R_{solution,\ A}$. and $R_{solution,\ B.}$ are the resistances of the experimental buffers, $R_{support}$ is the resistance of the support the cells are cultured on (the external circuit is similar to Figure 1, but is omitted from this diagram for clarity). It follows from the diagram that R_{total} is the sum of the resistances in the equation (where $R_{monolayer}$ lumps both the paracellular and transcellular resistance). In experimental practice, the resistances of the solutions and the permeable support are measured in a setup without cells and the measuring equipment is adjusted to a baseline correcting for the non-monolayer resistances. R_{total} will then be equivalent to $R_{monolayer}$. The $R_{monolayer}$ of endothelial or epithelial monolayers is called TransEndothelial (or Epithelial) Electrical Resistance, commonly abbreviated TEER.

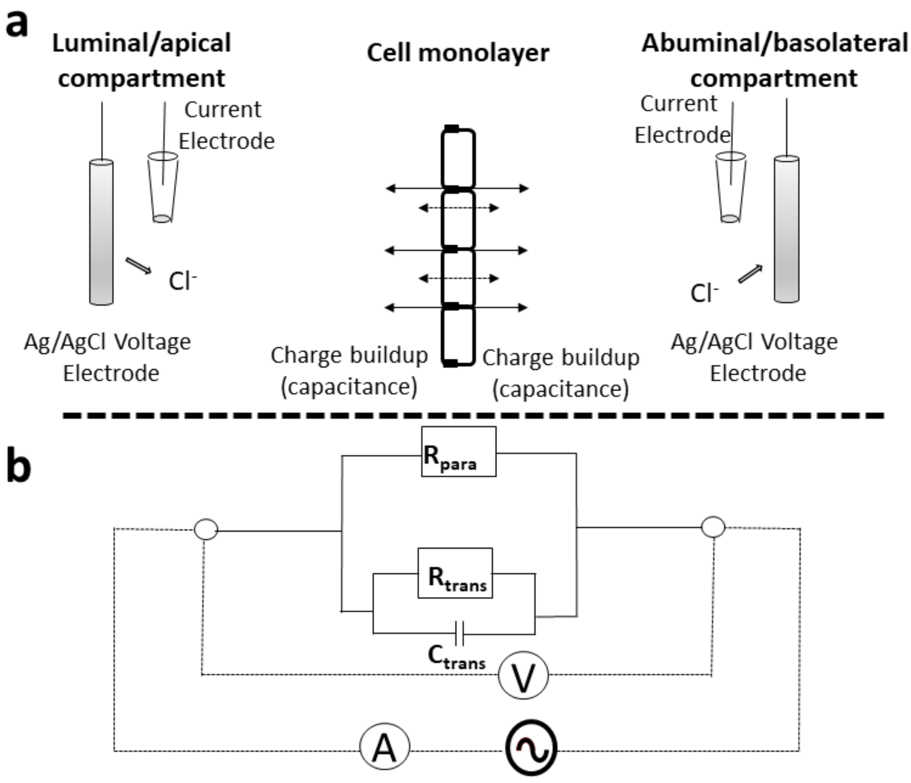

Figure S3. Schematic representation of contributors to impedance of a cell monolayer cultured in a two-compartment setup. a. The current can flow through the cell monolayer paracellularly or transcellularly. There will be a resistor formed by the tight junctions that tightly connect adherent cells together, and the lipid bilayers allowing the current to flow through the cell membrane will contribute to both an ohmic resistance in the membrane and an electrical capacitance. **b.** A diagram showing the resistors and capacitors in the system. $R_{paracellular,\ A}$ and $R_{transcellular}$ are the resistances of the cell monolayer, and will make the total monolayer resistance, $R_{monolayer}$. (In a situation where $R_{transcellular}$ is close to infinity, current will mainly flow across the capacitor and junctions, and the $R_{transcellular}$ can be ignored from the circuit diagram.)

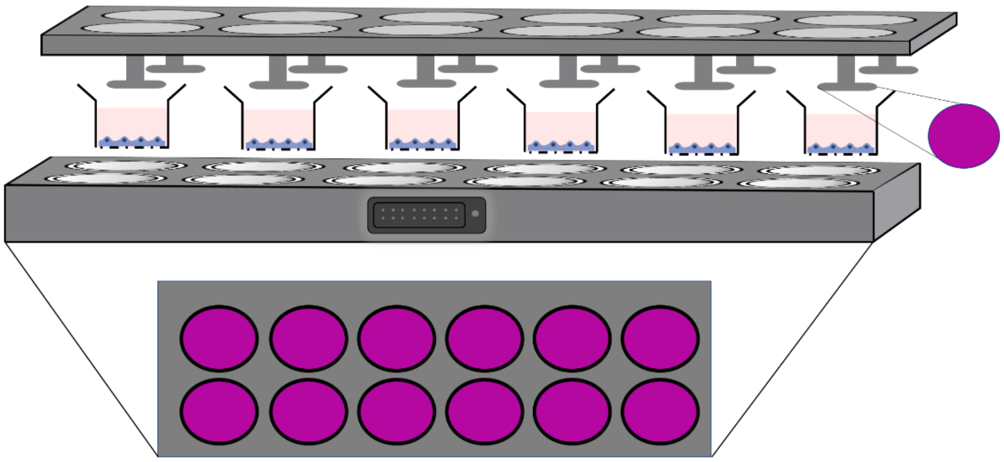

Figure S4. Schematic representation of the cellZscope system. The setup is based on electric impedance spectroscopy. Here the cell monolayers grown on cell culture inserts are between a pair of electrodes. During the measurement an alternating voltage is applied across the model and the amplitude and phase of the current is measured. With this device impedance spectra are automatically measured from which both resistance and capacitance data can be derived.

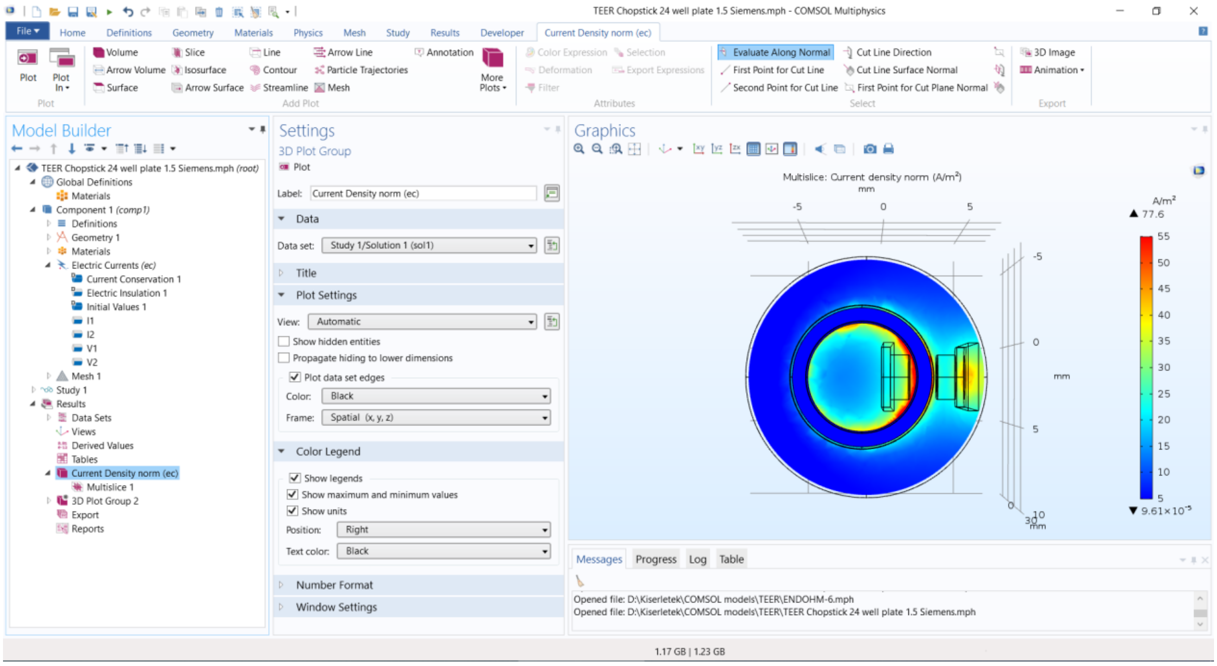

Figure S5. Screenshot of the graphical user interface (GUI) of the Comsol software. At left side the categories of editable parameters and parts are indicated, which are set for the simulation by the user. Among them the physics module is shown as well (Electric Currents etc.). The middle section shows the actual parameters of the selected part (marked by blue background). Results of this calculations are plotted and shown in the right window.

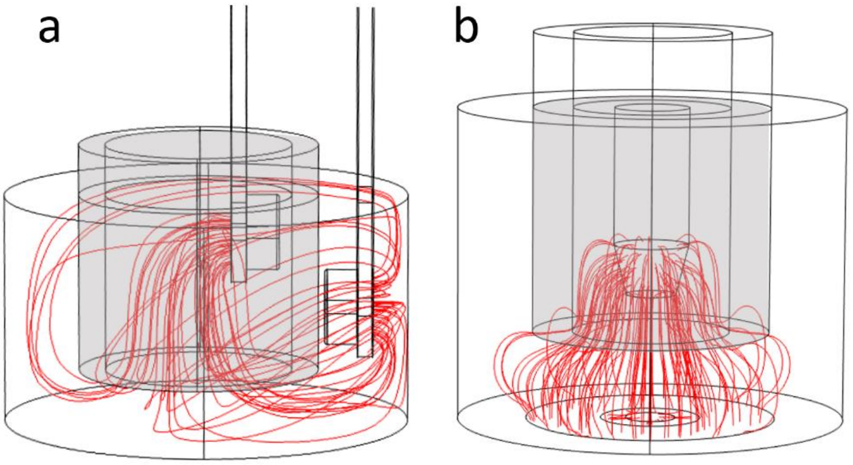

Figure S6. Comsol modeling of electrical resistance measurement across culture inserts (diameter: 6.5 mm, 24-well type) with **(a)** chopstick (STX2) or **(b)** chamber electrodes. The thin black lines show the borders of the objects and their components (borders of the electrodes on the surface). For easier understanding, the optically transparent cylindrical wall of the inserts is depicted in gray. The red lines show the current density in streamline representation.

References

Gerhartl A, Hahn K, Neuhoff A, Friedl HP, Förster CY, Wunder C, Schick M, Burek M, Neuhaus W. Hydroxyethylstarch (130/0.4) tightens the blood-brain barrier in vitro. *Brain Res.* 2020, *1727,* 146560. doi: 10.1016/j.brainres.2019.146560

Goldeman C, Andersen M, Al-Robai A, Buchholtz T, Svane N, Ozgür B, Holst B, Shusta E, Hall VJ, Saaby L, Hyttel P, Brodin B. Human induced pluripotent stem cells (BIONi010-C) generate tight cell monolayers with blood-brain barrier traits and functional expression of large neutral amino acid transporter 1 (SLC7A5). *Eur J Pharm Sci.* 2021, *156,* 105577. doi: 10.1016/j.ejps.2020.105577.

Lin GC, Leitgeb T, Vladetic A, Friedl HP, Rhodes N, Rossi A, Roblegg E, Neuhaus W. Optimization of an oral mucosa in vitro model based on cell line TR146. *Tissue Barriers.* 2020, *8,* 1748459. doi: 10.1080/21688370.2020.1748459.

Stebbins MJ, Wilson HK, Canfield SG, Qian T, Palecek SP, Shusta EV. Differentiation and characterization of human pluripotent stem cell-derived brain microvascular endothelial cells. *Methods.* 2016, *101,* 93-102. doi: 10.1016/j.ymeth.2015.10.016.
